# Acid Sphingomyelinase Downregulation Enhances Mitochondrial Fusion and Promotes Oxidative Metabolism in a Mouse Model of Melanoma

**DOI:** 10.3390/cells9040848

**Published:** 2020-03-31

**Authors:** Marco Coazzoli, Alessandra Napoli, Paulina Roux-Biejat, Clara De Palma, Claudia Moscheni, Elisabetta Catalani, Silvia Zecchini, Vincenzo Conte, Matteo Giovarelli, Sonia Caccia, Patrizia Procacci, Davide Cervia, Emilio Clementi, Cristiana Perrotta

**Affiliations:** 1 Department of Biomedical and Clinical Sciences “Luigi Sacco” (DIBIC), Università degli Studi di Milano, 20157 Milano, Italy; marco.coazzoli@unimi.it (M.C.); alessandra.napoli@unimi.it (A.N.); paulina.roux@unimi.it (P.R.-B.); claudia.moscheni@unimi.it (C.M.); silvia.zecchini@unimi.it (S.Z.); matteo.giovarelli@unimi.it (M.G.); sonia.caccia@unimi.it (S.C.); 2 Unit of Clinical Pharmacology, University Hospital “Luigi Sacco”-ASST Fatebenefratelli Sacco, 20157 Milano, Italy; 3 Department of Medical Biotechnology and Translational Medicine (BIOMETRA), Università degli Studi di Milano, 20129 Milano, Italy; clara.depalma@unimi.it; 4 Department for Innovation in Biological, Agro-food and Forest systems (DIBAF), Università degli Studi della Tuscia, 01100 Viterbo, Italy; ecatalani@unitus.it (E.C.); d.cervia@unitus.it (D.C.); 5 Department of Biomedical Sciences for Health (SCIBIS), Università degli Studi di Milano, 20133 Milano, Italy; vincenzo.conte@unimi.it (V.C.); patrizia.procacci@unimi.it (P.P.); 6 Scientific Institute IRCCS “Eugenio Medea”, 23842 Bosisio Parini, Italy

**Keywords:** melanoma, acid sphingomyelinase, mitochondrial dynamics, mitochondrial function

## Abstract

Melanoma is the most severe type of skin cancer. Its unique and heterogeneous metabolism, relying on both glycolysis and oxidative phosphorylation, allows it to adapt to disparate conditions. Mitochondrial function is strictly interconnected with mitochondrial dynamics and both are fundamental in tumour progression and metastasis. The malignant phenotype of melanoma is also regulated by the expression levels of the enzyme acid sphingomyelinase (A-SMase). By modulating at transcriptional level A-SMase in the melanoma cell line B16-F1 cells, we assessed the effect of enzyme downregulation on mitochondrial dynamics and function. Our results demonstrate that A-SMase influences mitochondrial morphology by affecting the expression of mitofusin 1 and OPA1. The enhanced expression of the two mitochondrial fusion proteins, observed when A-SMase is expressed at low levels, correlates with the increase of mitochondrial function via the stimulation of the genes PGC-1alpha and TFAM, two genes that preside over mitochondrial biogenesis. Thus, the reduction of A-SMase expression, observed in malignant melanomas, may determine their metastatic behaviour through the stimulation of mitochondrial fusion, activity and biogenesis, conferring a metabolic advantage to melanoma cells.

## 1. Introduction

Whereas the majority of malignant tumours rely on enhanced glycolysis for energy supply (i.e., the Warburg effect) [1], melanoma, the most aggressive form of skin cancer, has a unique metabolism, orchestrated by its environment and specific signalling mutations [2,3,4,5]. Although melanoma cells can rely on anaerobic metabolism, oxidative phosphorylation (OxPhos) also plays a role, which becomes critical in defined subsets of melanomas [6,7,8]. Increasing evidence indicates that mitochondrial respiration contributes to transformation, development of drug resistance and metastasis in melanomas, thus defining a pleiotropic role of mitochondria in tumourigenesis [9,10,11,12,13]. In normal and cancer cells, mitochondria exist in a dynamic network resulting from the interplay between fission and fusion events [14,15], governed by nutrient levels and energy demands [16]. In fragmented mitochondria, when fission exceeds fusion, oxidative metabolism is reduced and glycolytic intermediates are preserved for a highly activated glycolysis to provide fuel for cell proliferation [17,18]. In contrast, when mitochondrial fusion prevails, the outcome is an extension in the mitochondrial network, an event that provides specific metabolic advantages to cells under high energy needs, such as metastatic cells. The metabolic shift towards a more pronounced aerobic activity [18,19] also accounts for tumour resistance to drugs such as B-RAF inhibitors [20,21,22]. In mammalian cells, the primary players in mitochondrial morphology are mitofusin1 and mitofusin2 (Mfn1, Mfn2), and optic atrophy 1 (OPA1), which are essential for outer and inner mitochondrial membrane fusion, respectively, and dynamin-related protein 1 (Drp1), which is essential for the process of fission [14,15,19,23].

The malignant phenotype of melanoma, in terms of tumour progression and metastatic capacity, is also regulated by sphingolipids [24,25], in particular those originating from the activity of the lysosomal hydrolase acid sphingomyelinase (A-SMase), an enzyme responsible for the conversion of sphingomyelin to ceramide, a well-known pro-apoptotic molecule in both normal and cancer cells [26]. In melanoma cells, sphingolipid signalling is deregulated so as to prevent the accumulation of ceramide and protect cells from apoptosis [27,28]. One of the mechanism adopted by melanoma to promote cell proliferation and resistance to apoptosis is the downregulation of A-SMase expression and activity during tumour progression [29,30,31]. Although the role of mitochondria and A-SMase in melanoma share some similarities, and the localisation of A-SMase to mitochondria has been suggested [32], whether regulation by A-SMase of mitochondria explains its function in melanoma has never been studied.

Here, we report on a pivotal role of A-SMase in determining mitochondrial morphology and bioenergetics in a mouse model of melanoma cells. The reduction of A-SMase expression, reported to prompt progression of melanomas and influence their metastatic behaviour [31], leads to an increase of mitochondrial fusion, activity and biogenesis, thus conferring a selective metabolic advantage to melanoma cells.

## 2. Materials and Methods

### 2.1. Cell Models

The cell clones used in the in vivo experiments were generated from the murine melanoma cell line B16-F1 of the American Type Culture Collection. As described previously [31], the B16-W6_pSIL10 clone was generated from a subclone of the parental cell line B16-F1 (i.e., B16-W6) by transfecting the cells with the plasmid pSilencer4.1-CMV (Invitrogen-Life Technologies, Monza, Italy) containing a shRNA sequence responsible for A-SMase silencing. The control clone B16-pSILscr was generated by transfecting B16-W6 cells with the plasmid pSilencer4.1-CMV containing a shRNA scrambled sequence. The B16-B9 clone was also used as control of naturally occurring down-regulation of A-SMase.

For the in vitro experiments, we used B16-F1 cells transiently transfected with a A-SMase-specific (B16-F1_siASM: sense 5′-GGCUACCGAGUUUACCAAAtt-3′/antisense 5′-UUUGGUAAACUCGGUAGCCag-3′) or Mitf-specific (B16-F1_siMitf: sense: 5′-GGACAAUCACAACUUGAUUtt-3′/antisense: 5′-AAUCAAGUUGUGAUUGUCCtt-3′) siRNA or a non-targeting siRNA (B16-F1_scr: sense 5′-GCCTATGCTAATCGCAAATGTtt-3′/antisense 5′-ACAUUUGCGAUUAGCAUAGGCag-3′), alone or in combination. Briefly, B16-F1 cells were seeded at 40% confluence and transfected when at 60% confluence with the siRNAs using Lipofectamine RNAiMAX transfection reagent according to the manufacturer’s protocol for 48 h. B16-B1A cells, constitutively transfected with a the pEF1/Myc plasmid (Invitrogen-Life Technologies) containing the cDNA for A-SMase [30], were also used as a model of A-SMase overexpression.

Cells were cultured in Iscove’s modified Dulbecco’s medium supplemented with 10% heat-inactivated foetal bovine serum (FBS), glutamine (200 mM), and penicillin/streptomycin (100 U/mL), and grown at 37 °C in a humidified atmosphere containing 5% CO_2_.

### 2.2. Animal Handling and Allograft Tumour Model

Female C57BL/6 mice (6–8 weeks old) were purchased from Charles River Laboratories (Calco, Italy), housed in a regulated environment (23 ± 1 °C, 50% ± 5% humidity) with a 12 h light/dark cycle, and provided with food and water ad libitum. On day 0, mice were injected sub-cutaneously with a tumourigenic dose of 2.5 × 10^4^ melanoma cells in the lower-right flank [29,30,33,34]. Before the injection, melanoma cells were controlled for their levels of expression of A-SMase. Tumour growth was monitored every 2–3 days by means of a caliper and the volume was calculated (length × width 2/2). Mice were sacrificed when the tumour size reached ca. 500 mm^3^ volume; the tumour was then collected for transmission electron microscopy analyses. All studies were conducted in accordance with the Italian law on animal care N° 116/1992 and the European Communities Council Directive EEC/609/86. The experimental protocols (01/11) were approved by the Ethics Committee of the University of Milan. All efforts were made to reduce both animal suffering and the number of animals used.

### 2.3. Transmission Electron Microscopy

Samples collected from melanoma transplants were reduced into smaller blocks and fixed overnight at 4 °C in a solution containing 2% formaldehyde and 2% glutaraldehyde in 0.1 M sodium cacodylate buffer, pH 7.3. Specimens were washed in cacodylate buffer and postfixed at 0 °C for 1.5 h in 2% osmium tetroxide. The samples were washed in distilled water, stained en bloc in 2% aqueous uranyl acetate, dehydrated through an ascending series of ethanol, and embedded in Epon Araldite resin. For ultrastructural observations, at least 5 ultra-thin sections (60–90 nm) were obtained from each tumour. Sections were collected on 100-mesh grids, counterstained with lead citrate, and photographed (magnification 2500×) with an EM 10 electron microscope (Carl Zeiss, Oberkochen, Germany). Micrographs were scanned in a flat-bed scanner and images were merged.

For each melanoma transplant, at least 40,000 µm^2^ of each section area was analysed (Image ProPlus 6.0 software, Media Cybernetics, Bethesda, MD). Software was used to manually trace mitochondrial length, mitochondrial area and cytoplasm area of cells.

### 2.4. Immunofluorescence and Mitochondria Morphometric Analysis

B16-F1_siASM and B16-F1_scr cells, for a set of experiments, and B16-W6 and B16-B9 cells, for another, cultured in 120-mm coverslips were fixed in 4% paraformaldehyde in 0.1 M phosphate buffer (PB), pH 7.4, for 10 min. Samples were then washed in PB and pre-incubated for 30 min at room temperature with 5% bovine serum albumin (BSA; Life Technologies, Monza, Italy) and 10% of normal goat serum (Life Technologies) in PB containing 0.1% Triton X-100. Subsequently, samples were stained overnight at 4 °C with mouse anti-cyclophilin f (#ab110324; Abcam, Cambridge, UK) primary antibody at a dilution of 1:200 in PB containing 0.1% Triton X-100. Cells were then stained for 1 h at room temperature with the appropriate Alexa Fluor secondary antibody with or without the cytopainter phalloidin-ifluor 555 (#ab176756; Abcam) that binds to F-actin filaments (cytoskeleton detection). Finally, samples were cover-slipped in a ProLong Gold Antifade Mountant with or without DAPI (Life Technologies). Images were acquired with a Zeiss LSM 710 confocal microscope (Carl Zeiss) [35,36].

To quantify mitochondrial morphology, an ImageJ macro was used [37]. In brief, acquired images were converted to binary and the signal from cyclophylin (green) was optimised to a threshold allowing resolution of individual mitochondria. The macro “analyze particles” was applied to detect mitochondrial outlines and quantify both mitochondrial size and interconnectivity for each mitochondrion. The mean area/perimeter ratio was used as an index of mitochondrial interconnectivity where high values indicate mitochondria with many physical interactions, while low values are indicative of single mitochondria. For the analysis of mitochondrial branching, the binary image was converted to a skeleton by using the “skeletonize” plugin. Finally, the length of each branch and the number of branches were determined by using the “analyze skeleton” plugin [38].

### 2.5. Quantitative Real Time-PCR (qPCR)

The analysis of mRNA expression was performed as previously described [31,33,35,39,40]. Briefly, total RNA from cells was extracted with the PureZol RNA Isolation Reagent (Bio-Rad, Hercules, CA, USA), according to the manufacturer’s protocol. First-strand cDNA was generated from 1 μg of total RNA using the iScript Reverse Transcription Supermix (Bio-Rad, Hercules, CA, USA). A set of primer pairs (Eurofins Genomics, Milan, Italy) was designed to hybridise to unique regions of the appropriate gene sequence (Table 1). qPCR was performed using the SsoAdvanced Universal SYBR Green Supermix and the CFX96 Touch Real-Time PCR Detection System (Bio-Rad, Hercules, CA, USA). The fold change was determined relative to the control after normalisation to internal standards (actin, rpl32, 36b4) through the use of the formula 2^−ΔΔCT^. Mitochondrial DNA (mtDNA) was quantified as previously described [41,42]. Total DNA was isolated from cells using the QIAamp DNA mini kit (Qiagen, Milano, Italy) according to the protocol provided by the manufacturer. The mtDNA content was measured by quantitative PCR, normalising the quantity of a not-polymorphic mtDNA with a single copy nuclear gene (RNAse P).

### 2.6. Protein Isolation and Western Blotting

Mouse melanoma cells were lysed for 10 min at 4 °C in a lysis buffer containing 50 mM Tris-HCl pH 7.4, 150 mMNaCl, 1% NP40, 0.25% Na-deoxycholate and supplemented with a cocktail of protease and phosphatase inhibitors (cOmplete and PhosSTOP; Roche Diagnostics, Milano, Italy). Equal amounts of proteins (40 μg/lane) were separated by 4–20% SDS-polyacrylamide gel electrophoresis (Criterion TGX Stain-free precast gels and Criterion Cell system; Bio-Rad, Hercules, CA, USA). Proteins were then transferred onto nitrocellulose membrane using a Bio-Rad Trans-Blot Turbo System [36,43,44]. The membranes were probed using the following primary antibodies: Mouse monoclonal anti-Mfn1 (#H00055669-M04, Abnova, Taipei City, Taiwan), anti-OPA1 (#612606, Becton Dickinson, Franklin Lakes, NJ, USA) and anti-vinculin (#v4505, Sigma-Aldrich, Saint Louis, MO, USA) as loading control. After the incubation with the appropriate horseradish-peroxidase (HRP)-conjugated secondary antibody (Bio-Rad, Hercules, CA, USA), bands were visualised using the Clarity Western ECL substrate with a ChemiDoc MP imaging system (Bio-Rad, Hercules, CA, USA). Bands were quantified for densitometry using the Image Lab software (Bio-Rad, Hercules, CA, USA) [29,34,45,46].

### 2.7. Mitochondria Respiratory Rate

Mitochondria respiratory rates were measured into the O2K oxygraph chambers (Oroboros Instruments, Innsbruck, Austria) at 37 °C in the respiration medium MiR06 (0.5 mM EGTA, 3 mM MgCl2, 60 mM K-lactobionate, 20 mM taurine, 10 mM KH2PO4 20 mM Hepes, 110 mM sucrose and 1 g/L bovine serum albumin fatty acid-free, 280 U/mL catalase (pH 7.1)) [37,41,42,47]. Basal respiration was observed when the signal of oxygen consumption was stable, while the leak respiration was induced after the addition of oligomycin (0.5 µM). Stepwise titration (0.5 μM each step) of the uncoupler carbonyl cyanide-4-(trifluoromethoxy) phenylhydrazone (FCCP) induced the progressive release of the proton gradient until maximal respiration was achieved. The residual oxygen consumption was evaluated by blocking mitochondrial respiration with the addition of 0.5 μM rotenone and 2.5 μM antimycin A (AA), and this value was subtracted by each steady-state. Coupling efficiency was the ratio: (Basal Respiration - Leak respiration)/Basal respiration.

### 2.8. ATP Production

ATP concentration was measured by the luciferin-luciferase assay [41,42]. Briefly, cells (10^6^ cells) were permeabilised with digitonin (10µg/10^6^ cells) in buffer-A (150mM KCl, 25mMTris-HCl, 2mM EDTA, 0.1% BSA, 10mM potassium phosphate and 0.1mM MgCl2 (pH 7.4)) at room temperature with gentle agitation. Permeabilised cells were plated in 96 wells (2 × 10^5^ cells/well) and treated with a mix containing 2 mM malate, 1 mM pyruvate, 1 mM ADP and buffer-B (0.2 mM luciferin and 5 μg/mL luciferase in 0.5M Tris-acetate (pH 7.75)). Oligomycin (2 µg/mL) was also added to detect glycolytic ATP in our samples and ATP was measured using a GloMax luminometer (Promega, Madison, WI, USA)

### 2.9. Mitochondrial Membrane Potential Analysis

Mitochondrial potential was measured by cell staining with 500 nM of the sensitive fluorescent dye, TMRM (Sigma-Aldrich, Saint Louis, MO, USA), as previously described [33,48,49]. Fluorescence was analysed by a Gallios Flow Cytometer (Beckman-Coulter, Brea, CA, USA) and the software FCS Express 4 (De Novo System, Portland, OR, USA).

### 2.10. Statistical Analysis

Statistical significance of differences between the groups was evaluated using Student’s t-test (single comparisons) or one-way ANOVA, followed by the Newman–Keuls post-test (multiple comparisons). When data were not normally distributed and the variance between the samples differed significantly, the Mann–Whitney test or the Wilconox test was used. Data belonging from different experiments were represented and averaged in the same graph. The GraphPad Prism software package (Graph Software, San Diego, CA, USA) was used. The results were expressed as means ± SEM of the indicated n values.

## 3. Results

### 3.1. A-SMase Expression Determines Mitochondrial Morphology

**Figure 1:**
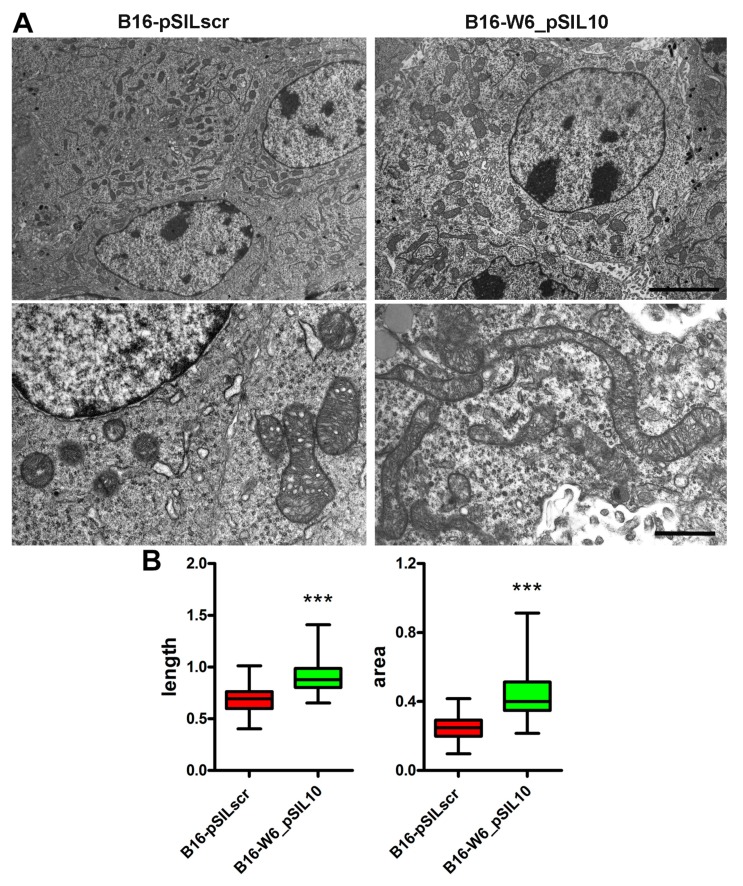
A-SMase expression determines mitochondrial morphology in vivo. C57BL/6 mice (*n* = 3) were injected in the right flank with B16_pSILscr and B16-W6_pSIL10 cells; tumours were then resected when they reached a volume of 500 mm^3^. (**A**) Transmission electron microscopy showing mitochondria in B16_pSILscr and B16-W6_pSIL10 tumours. In B16-pSILscr, mitochondria appear smaller and round in shape. In B16-W6_pSIL10, mitochondria appear rather elongated and with a larger area. Upper panels scale bar = 5 μm. Lower panels scale bar = 1 μm. (**B**) Blot-and-whisker plot showing the quantification of mitochondria length (left graph) and area (right graph) per unit of surface area (100 μm^2^). Statistical significance *** *p* < 0.001 vs. B16_pSILscr.

Depending on the cell type and physiological conditions, mitochondria can be present either as numerous morphologically distinct small organelles, or they can form large interconnected networks [14,15]. To assess the relationship between A-SMase expression and mitochondrial morphology, we used transmission electron microscopy to analyse the shape of the mitochondria of explanted murine melanoma allografts. To this end, mice were injected subcutaneously with B16-W6_pSIL10 cells, a cell clone obtained by constitutively knocking-down A-SMase in B16-F1 cells [29,30,31]. B16-pSILscr cells transfected with the scrambled vector were used as a control. By ultrastructural analysis we found that control tumours presented mostly rounded and small mitochondria, while elongated and more tubular mitochondria accumulated in B16-W6_pSIL10 melanomas (Figure 1A). The measurement of the mitochondria mean length and area confirmed that these organelles were significantly longer and larger in the absence of A-SMase (Figure 1B). The effect of A-SMase expression levels on mitochondrial shape was also confirmed in allograft melanomas derived from the B16-B9 clone, in which A-SMase is expressed naturally at low levels. In these tumours, mitochondria appear round, rather elongated in shape, and with a larger area (Supplementary Figure S1A).

### 3.2. A-SMase Expression Regulates Mitochondrial Elongation through Mfn1 and OPA1

**Figure 2:**
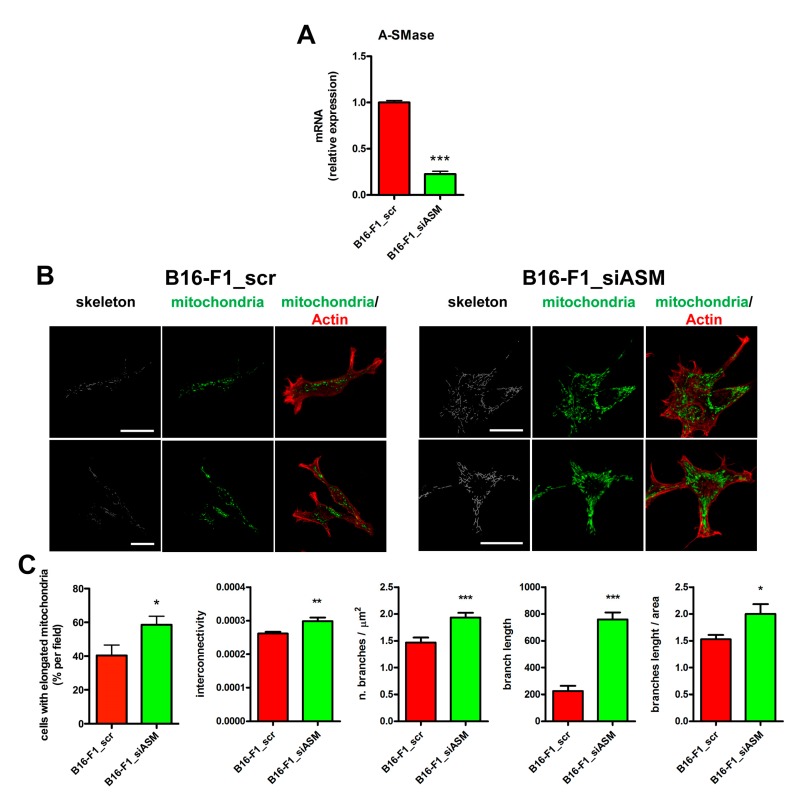
A-SMase expression regulates mitochondrial elongation in vitro. B16-F1 cells were transiently transfected with the scrambled siRNA (B16-F1_scr) or with an A-SMase siRNA (B16-F1_siASM). (**A**) A-SMase expression was evaluated by qPCR (*n* ≥ 6). Data are expressed as fold change over B16-F1_scr. *** *p* < 0.001 vs. B16-F1_scr. (**B**) Representative fluorescence micrographs and skeleton images of cyclophylin f and actin staining of B16-F1_scr and B16-F1_siASM cells. Scale bar = 20 µm. (**C**) Percentage of cells with elongated mitochondria, mitochondrial interconnectivity, number of branches, branch length and branch length/area are shown in the graphs. * *p* < 0.05; ** *p* < 0.01; *** *p* < 0.001 vs. B16-F1_scr.

Given our initial observation, we aimed to determine whether the differences in mitochondrial size observed in explanted tumours (Figure 1A,B) depended on A-SMase expression and, if so, the mechanism behind this event. To this end, we analysed in vitro the effect of A-SMase silencing on mitochondrial morphology by transiently transfecting B16-F1 cells with a siRNA specific for A-SMase (B16-F1_siASM cells) (Figure 2A) [31]. We found that the downregulation of A-SMase resulted in an increased percentage of cells with elongated mitochondria which were characterised by augmented interconnectivity, number of branches and branch length compared to scrambled control (B16-F1_scr) (Figure 2B,C). These results are in line with those obtained in the two clones derived from B16-F1 cells expressing A-SMase at low (B19-B9) and high levels (B16-W6). B19-B9 cells displayed a mitochondrial network with elongated mitochondria, similar to that observed in B16-F1_siASM cells, while B16-W6 showed more rounded mitochondria (Supplementary Figure S1B). All these data confirm further that A-SMase expression affects mitochondrial morphology.

**Figure 3:**
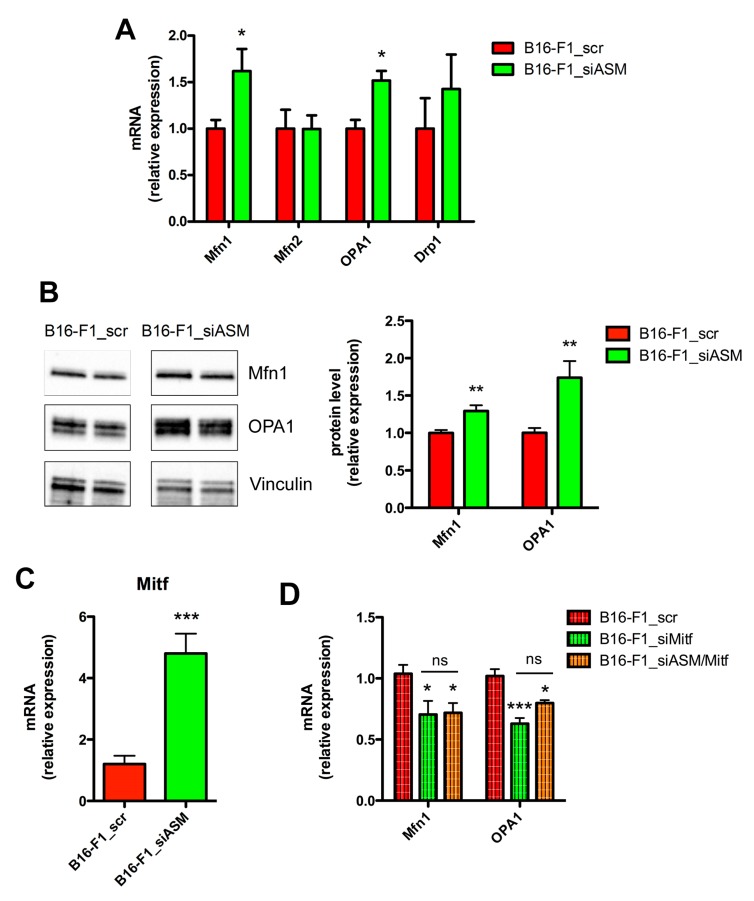
A-SMase downregulation enhances the expression of Mfn1 and OPA1. (**A**) qPCR of Mfn1, Mfn2, OPA1 and Drp1 on mRNA extract from B16-F1_scr and B16-F1_siASM cells (*n* = 6). Data are expressed as fold change over B16-F1_scr. * *p* < 0.05 vs. B16-F1_scr. (**B**) Western blotting of Mfn1, OPA1 and Vinculin (loading control) on B16-F1_scr and B16-F1_siASM cells. Images shown on the left are representative of one out of three reproducible experiments. Right panels: densitometric analysis of Mfn1 and OPA1 normalised on Vinculin. ** *p* < 0.01 vs. B16-F1_scr. (**C**) qPCR of Mitf on mRNA extract from B16-F1_scr and B16-F1_siASM cells (*n* ≥ 6). Data are expressed as fold change over B16-F1_scr. *** *p* < 0.001 vs. B16-F1_scr. (**D**) qPCR of, Mfn1 and OPA1 on mRNA extract from B16-F1_scr and B16-F1_siMitf and B16-F1_siASM/Mitf cells (*n* ≥ 6). * *p* < 0.05; *** *p* < 0.001 vs. B16-F1_scr.

The balance of mitochondrial fusion and fission dictates the morphology, abundance, function and spatial distribution of mitochondria. Therefore, we analysed the expression of the players of mitochondrial fusion, i.e., Mfn1, Mfn2 and OPA1 and fission i.e., Drp1 [14,15,19,23]. We found that the expression of Mfn1 and OPA1 at both the mRNA and protein level increased significantly in B16-F1_siASM cells, while no differences were observed for the mRNA of Mfn2 and Drp1 (Figure 3A,B). On the contrary, the analysis of Mnf1 and OPA1 in a clone overexpressing A-SMase (B16_B1A) showed that the increase of A-SMase expression induced a reduction of the two markers of mitochondrial fusion (Supplementary Figure S1C).

To better understand this mechanism, we investigated whether the microphtalmia-associated transcription factor (Mitf), a key target of A-SMase action in melanoma [31], was involved in mitochondrial dynamics and their changes. As shown in Figure 3C, we found increased levels of Mitf mRNA in B16-F1_siASM cells when compared to B16-F1_scr cells (Figure 3C), further confirming its dependency on A-SMase expression. Notably, the silencing of Mitf (Figure 3D) in B16-F1 cells induced a significant inhibition of Mfn1 and OPA1 mRNA expression (Figure 3D), therefore acting, as expected, in an opposite manner to A-SMase. To further explore the Mitf-dependency in the context of the A-SMase pathway, we silenced A-SMase and Mitf together in B16-F1 cells (B16-F1_siASM/siMitf) (Supplementary Figure S2A,B), finding that silencing Mitf completely abolished the effect of A-SMase down-regulation on Mfn1 and OPA1 expression (Figure 3D).

Taken together, these data indicate that low levels of A-SMase expression, as reported to occur in melanoma [31], are a key determinant of mitochondrial morphology, acting by increasing mitochondrial fusion through Mitf upregulation.

### 3.3. A-SMase Downregulation Improves Mitochondrial Function

Mitochondrial dynamics are regulated by cellular bioenergetic demands. Mitochondria are the major source of ATP and metabolites necessary to fulfill the bioenergetics and biosynthetic requirements of cells [50,51]. Thus, we evaluated whether the changes in mitochondrial morphology following A-SMase silencing might affect mitochondrial activity.

**Figure 4:**
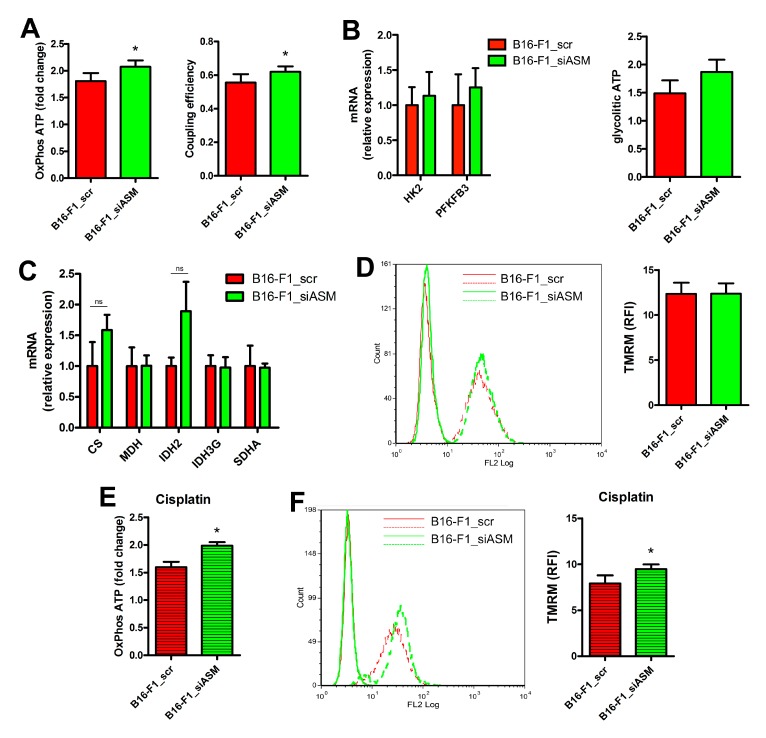
A-SMase expression regulates mitochondrial function. (**A**) Measurement of ATP production through oxidative phosphorylation by mitochondria (at 10 min after substrate addition and normalised on the value at time 0) and coupling efficiency of B16-F1_scr and B16-F1_siASM cells (*n* = 3). * *p* < 0.05 vs. B16-F1_scr. (**B**) Left panel: qPCR of HK2 and PFKFB3 on mRNA extract from B16-F1_scr and B16-F1_siASM cells (*n* ≥ 3). Data are expressed as fold change over B16-F1_scr. Right panel: Measurement of ATP production through glycolysis in B16-F1_scr and B16-F1_siASM cells. Values are expressed as the ATP produced at 10 min after substrate addition and normalised on the value of ATP at time 0 (*n* = 4). (**C**) qPCR of CS, MDH, IDH2, IDH3G and SDHA on mRNA extract from B16-F1_scr and B16-F1_siASM cells (*n* ≥ 3). Data are expressed as fold change over B16-F1_scr. (**D**) Evaluation by flow cytometry of mitochondrial membrane potential. Left panel: Histograms of TMRM staining of B16-F1_scr and B16-F1_siASM cells. Right panel: Quantification of TMRM staining by measurement of RFI (*n* = 4). (**E**) Measurement of ATP production through oxidative phosphorylation by mitochondria of B16-F1_scr and B16-F1_siASM cells treated with Ciplatin (10 μg/mL for 16 h). Values are expressed as the ATP produced at 10 min after substrate addition and normalised on the value of ATP at time 0, *n* = 3, * *p* < 0.05 vs. B16-F1_scr. (**F**) Left panel: Histograms of TMRM staining of B16-F1_scr and B16-F1_siASM cells treated with Cisplatin. Right panel: quantification of TMRM staining by measurement of RFI (*n* = 4), * *p* < 0.05 vs. B16-F1_scr.

In B16-F1_siASM cells, we found an increased production of ATP via OxPhos, sustained by an improved mitochondrial coupling efficiency (Figure 4A). This event depends on the activation of the respiratory chain and not on upstream biochemical pathways. Indeed, no differences were observed in the expression of genes encoding for proteins involved in glycolysis and Krebs cycles, and no modifications were detected in the production of glycolytic ATP (Figure 4B,C). No changes were noticed in the mitochondrial membrane potential of B16-F1_siASM compared to B16-F1_scr, measured by loading the cells with the potentiometric probe tetramethylrhodamine methyl ester (TMRM) [33] (Figure 4D).

A-SMase silencing reduces the sensitivity to Cisplatin of B16 melanoma cells [29]. Therefore, we assessed whether mitochondria could be involved in melanoma cell response to the chemotherapeutic drug. Cisplatin (10 μg/mL for 16 h) enhanced OxPhos ATP production and mitochondrial membrane potential in B16-F1_siASM cells compared to B16-F1_scr (Figure 4E,F). This indicates that A-SMase has a role in the cytotoxic response to Cisplatin, and that this goes through the activation of mitochondrial pathways.

### 3.4. A-SMase Downregulation Increases Mitochondrial Biogenesis

The increase of mitochondrial mass by mitochondrial biogenesis is a mechanism that promotes metastasis and resistance to chemotherapy in different cancers [9,52,53,54].

**Figure 5:**
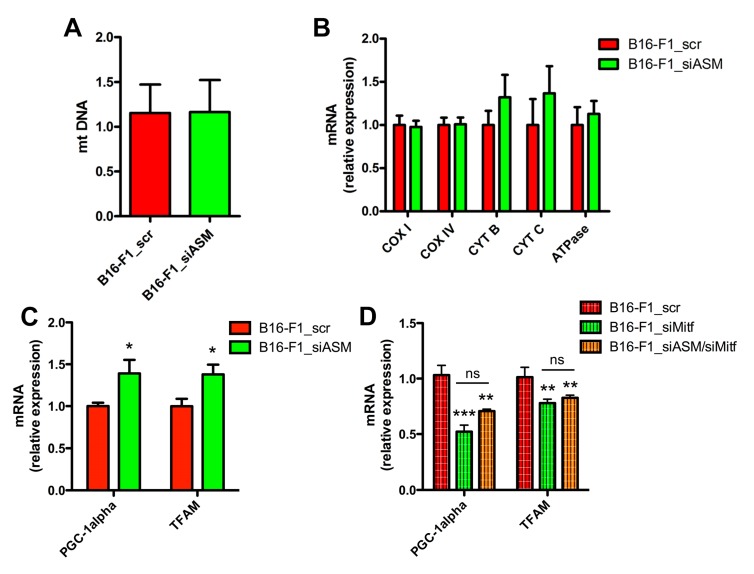
A-SMase downregulation increases mitochondrial biogenesis. (**A**) Analysis of mtDNA of B16-F1_scr and B16-F1_siASM cells (*n* = 6). (**B**) qPCR of COX I, COX IV, CYT B, CYT C and ATPase on mRNA extract from B16-F1_scr and B16-F1_siASM cells (*n* = 4). Data are expressed as fold change over B16-F1_scr. (**C**) qPCR of PGC-1alpha and TFAM on mRNA extract from B16-F1_scr and B16-F1_siASM cells (*n* ≥ 5). Data are expressed as fold change over B16-F1_scr. * *p* < 0.05 vs. B16-F1_scr. (**D**) qPCR of PGC-1alpha and TFAM on mRNA extract from B16-F1_scr and B16-F1_siMitf and B16-F1_siASM/Mitf cells (*n* ≥ 5). ** *p* < 0.01, *** *p* < 0.001 vs. B16-F1_scr.

The silencing of A-SMase did not lead to changes in mitochondrial content, measured via the analysis of mitochondrial DNA (mtDNA) in B16-F1_scr and B16-F1_siASM cells (Figure 5A). This datum was confirmed by the expression of genes encoding different electron transport chain subunits, namely, COX subunit I and IV (COX I and COX IV), cytochrome b and c (CYT B and CYT C), and ATPase (Figure 5B). However, when we analysed the expression of the major genes involved in the mitochondrial biogenesis machinery, we found in A-SMase silenced cells a higher level of mRNA of the peroxisome proliferator activated receptor-gamma co-activator 1 alpha (PGC-1alpha), as well as one of its downstream targets, the mitochondrial transcription factor A (TFAM) (Figure 5C). Of note, Mitf silencing alone or in combination with the downregulation of A-SMase in B16-F1 cells reduces the expression of both PGC-1alpha and TFAM (Figure 5D), indicating that ASMase levels affect the mitochondrial biogenesis system through the regulation of Mitf.

## 4. Discussion

The therapeutic potential of targeting sphingolipid metabolism in melanoma, a tumour for which a resolutive cure is still lacking and with a high risk of local or disseminated recurrence after surgical excision of the primary tumour [55], has been previously demonstrated [24,27,56,57,58]. Notably, some studies showed the involvement of A-SMase in critical phases of melanoma development such as proliferation, migration, the ability to metastasise, and the response to chemotherapy [29,30,31,59,60,61,62,63].

In the current study, we evaluated for the first time the role of A-SMase in the regulation of mitochondrial function and morphology in a mouse model of melanoma cells. We discovered that the A-SMase expression level is correlated with mitochondrial elongation, which is concurrent with an increased oxidative phosphorylation and the activation of the biogenesis machinery.

We found that A-SMase impacts mitochondrial elongation by regulating the expression of mitochondrial fusion proteins. A-SMase silencing promoted mitochondrial fusion by enhancing the expression of Mfn-1 and OPA1. A recent study showed that expression levels of the proteins involved in mitochondrial fusion and fission differ among melanoma cells [19]. In particular, this study revealed that inhibiting mitochondrial fusion in human melanoma cells significantly decreases oxygen consumption rate, thus indicating that oxidative phosphorylation depends on mitochondrial fusion in melanoma cells.

High expression of Mfn1, Mfn2 and OPA1 has also been linked to cancer cell proliferation, survival and invasion, while their inhibition blocks cell growth and triggers apoptosis of different cancer cells. Conversely, mitochondrial fission occurs during apoptosis and seems important for progression of the apoptotic pathway [17,64,65,66,67].

Mitochondrial dynamics and function are tightly interconnected; in order to satisfy the metabolic requirements of the cell, mitochondria constantly divide, elongate and connect with each other to form tubular networks or fragmented granules [17,68]. The analysis of melanoma cell lines and patient samples indicated that in some subsets of melanoma OxPhos plays a critical role [6,7,8], as between 35–50% of them show a “High-OxPhos” phenotype, characterised by increased mitochondrial respiration. This phenotype is, first and foremost, dictated by PGC-1alpha. The high expression of PGC-1alpha correlates with increased expression of mitochondrial transcriptional factors and mitochondrial fusion and fission mediators [13], together with a decreased overall survival in patients with stage III melanoma [7]. The “High-OxPhos” phenotype of melanoma cells corroborates what we observed in A-SMase-silenced B16-F1 cells, in which we found an increased production of OxPhos ATP and the activation of the mitochondrial biogenesis machinery. Notably, we did not notice differences between the control and the silenced cells in the content of mitochondrial DNA, a proxy for mitochondrial mass. However, the mitochondrial mass is determined by the balance of organelle biogenesis and degradation. We already demonstrated that autophagy in melanoma cells relies upon A-SMase expression levels and that low levels of A-SMase increase the autophagic process [29,69]. The fact that A-SMase deficiency enhances mitochondrial biogenesis machinery but fails to increment mitochondrial content may conceivably be through the activation of autophagy.

A-SMase signalling in melanoma involves the transcription factor Mitf [31]. A-SMase promotes the proteasomal degradation of Mitf, such that its levels increase whenever the levels of A-SMase are reduced, such as during melanoma development [31]. Here, we demonstrate that A-SMase regulates Mitf also at transcriptional levels, with the downregulation of Mitf resulting in the inhibition of Mfn-1 and OPA1 mRNA. The effect of Mitf on the expression of the two proteins may occur through a direct interaction with their promoters, or may involve the activation/inhibition of other molecular players. The analysis of the promoters of Mfn1 and OPA1 (*Mus musculus*), carried out by using the EPD database (https://epd.epfl.ch//index.php) [70], showed multiple putative consensus sequences for Mitf with both genes (Mfn1: - [*p*-value = 0.01]: -932, -931; -903, -902; -741, -740; -635, -634; -599, -598; -567, -566; -476, -475; -263, -262; -144, -143; -8, -7 to transcription start site (TSS); OPA1: [*p*-value = 0.01]: -912, -911; -442, -441; -337, -336; -273, -272; -85, -84; 18, 19 to TSS). Thus, the effect of Mitf on the expression of these two genes may occur through a direct interaction with their promoters, although the involvement of other molecular players cannot be excluded.

Mitf controls the expression of genes crucial for melanoma cell differentiation, proliferation, and survival [71,72]. The functional roles of Mitf have now been broadened by the identification of this transcription factor, together with PGC-1alpha, as a marker for melanoma subtypes that depend on mitochondrial OxPhos [8]. Melanomas present heterogeneous metabolic and energetic states, which are defined by the levels of expression of PGC-1alpha. PGC-1alpha-negative melanoma cells have a reduced bioenergetic capacity but high rates of glycolysis consistent with a glycolytic ‘‘Warburg’’ state. Conversely, PGC-1alpha-positive cells show elevated rates of mitochondrial oxidative metabolism [7]. Mitf expression sustains the transcription of PGC-1alpha in melanoma cells [7]. In our cell model, A-SMase downregulation induced the increase of Mitf and the “High OxPhos” phenotype; the silencing of the transcription factor correlated with the downregulation of PGC-1alpha and of its downstream target TFAM, whose expression has been correlated recently with glucose consumption and ATP production in melanoma cells [73].

In summary, our study demonstrates that A-SMase downregulation modulates the following: (i) mitochondrial morphology, by enhancing the expression of Mfn1 and OPA1; and (ii), mitochondrial biogenesis and function, through the stimulation of PGC-1alpha and TFAM. Moreover, we demonstrate that A-SMase acts on the two above biological events by modulating the expression of the mitochondrial fusion machinery and biogenesis in a Mitf-dependent manner. Our findings, therefore, expand the understanding of A-SMase in melanomas and provide new insights into its diverse roles in shaping the melanoma phenotype.

## Figures and Tables

**Table 1:** List of primers designed for PCR.

	Gene Accession Number	Primer Sequence	Amplicon
A-SMase (smpd1)	NM_011421	F: 5′-TGGGACTCCTTTGGATGGG-3′ R: 5′-CGGCGCTATGGCACTGAAT-3′	134 bp
Mfn1	NM_024200	F: 5′-CCTACTGCTCCTTCTAACCCA-3′ R: 5′-AGGGACGCCAATCCTGTGA-3′	86 bp
Mfn2	NM_133201	F: 5′-AGAACTGGACCCGGTTACCA-3′ R: 5′-CACTTCGCTGATACCCCTGA-3′	82 bp
OPA1	NM_133752	F: 5′-TGGAAAATGGTTCGAGAGTCAG-3′ R: 5′-CATTCCGTCTCTAGGTTAAAGCG-3′	76 bp
Drp1	NM_152816	F: 5′-GCTGGATCACGGGACAAGTTAA-3′ R: 5′-TGCCTGTTGTTGGTTCCTGAC-3′	106 bp
Mitf	NM_001113198 NM_008601 NM_001178049	F: 5′-CCAACAGCCCTATGGCTATGC-3′ R: 5′-CTGGGCACTCACTCTCTGC-3′	99 bp
HK2	NM_013820	F: 5′-TGATCGCCTGCTTATTCACGG-3′ R: 5′-AACCGCCTAGAAATCTCCAGA-3′	112 bp
PFKFB3	NM_001177752	F: 5′-CCCAGAGCCGGGTACAGAA-3′ R: 5′-GGGGAGTTGGTCAGCTTCG-3′	88 bp
CS	NM_026444	F: 5′-GGACAATTTTCCAACCAATCTGC-3′ R: 5′-TCGGTTCATTCCCTCTGCATA-3′	109 bp
MDH	NM_008617	F: 5′-TTGGGCAACCCCTTTCACTC-3′ R: 5′-GCCTTTCACATTTGCTCTGGTC-3′	131 bp
IDH2	NM_173011	F: 5′-GGAGAAGCCGGTAGTGGAGAT-3′ R: 5′-GGTCTGGTCACGGTTTGGAA-3′	139 bp
IDH3G	NM_008323	F: 5′-GGTGCTGCAAAGGCAATGC-3′ R: 5′-TATGCCGCCCACCATACTTAG-3′	136 bp
SDHA	NM_023281	F: 5′-GGAACACTCCAAAAACAGACCT-3′ R: 5′-CCACCACTGGGTATTGAGTAGAA-3′	106 bp
COX I	NC_005089.1	F: 5′-CCAGTGCTAGCCGCAGGCAT-3′ R: 5′-GCTGGTAGAGAATTGGGTCCCCTCC-3′	100 bp
COX IV	NM_009941	F: 5′-TACTTCGGTGTGCCTTCGA-3′ R: 5′-TTAGCATGGACCATTGGATACGG-3′	110 bp
CYT B	NC_005089.1	F: 5′-ACGCCATTCTACGCTCAATC -3′ R: 5′-GCTTCGTTGCTTTGAGGTAT-3′	110 bp
CYT C	NM_007808	F: 5′-ATAGGGGCATGTCACCTCAAAC-3′ R: 5′-GTGGTTAGCCATGACCTGAAAG-3′	172 bp
ATPase	NM_016774	F: 5′-CGTGAGGGCAATGATTTATACCAT-3′ R: 5′-TCCTGGTCTCTGAAGTATTCAGCAA-3′	170 bp
mtDNA	NC_005089	F: 5′-CCTATCACCCTTGCCATCAT-3′ R: 5′-GAGGCTGTTGCTTGTGTGAC-3′	194 bp
RNase P (DNA)	NC_000085	F: 5′-GAAGGCTCTGCGCGGACTCG-3′ R: 5′-CGAGAGACCGGAATGGGGCCT-3′	119 bp
PGC-1alpha	NM_008904	F: 5′-ACTATGAATCAAGCCACTACAGAC-3′ R: 5′-TTCATCCCTCTTGAGCCTTTCG-3′	143 bp
TFAM	NM_009360	F: 5′-AAGACCTCGTTCAGCATATAACATT-3′ R: 5′-TTTTCCAAGCCTCATTTACAAGC-3′	104 bp
36b4	NM_007475	F: 5′-AGGATATGGGATTCGGTCTCTTC-3′ R: 5′-TCATCCTGCTTAAGTGAACAAACT-3′	143 bp
RPL32	NM_172086	F: 5′-TTAAGCGAAACTGGCGGAAAC-3′ R: 5′-TTGTTGCTCCCATAACCGATG-3′	100 bp
Actin beta	NM_007393	F: 5′-GGCTGTATTCCCCTCCATCG-3′ R: 5′-CCAGTTGGTAACAATGCCATGT-3′	154 bp

## Supplementary Materials

The following are available online at https://www.mdpi.com/2073-4409/9/4/848/s1, Figure S1: A-SMase expression determines mitochondrial shape in vivo and in vitro, Figure S2: Control of A-SMase and Mitf double knockdown, Figure S3: Uncropped western blotting of the images shown in Figure 3B.
