# Supplementary material for: Acid Sphingomyelinase Downregulation Enhances Mitochondrial Fusion and Promotes Oxidative Metabolism in a Mouse Model of Melanoma
Source: Cells. 2020 Mar 31;9(4):848. doi: 10.3390/cells9040848 (PMC7226741; doi:10.3390/cells9040848)
Supplement: Supplementary file 1 [file cells-09-00848-s001.pdf]

Supplementary Material: Acid sphingomyelinase downregulation enhances mitochondrial fusion and promotes oxidative metabolism in a mouse model of melanoma

Marco Coazzoli, Alessandra Napoli, Paulina Roux-Biejat, Clara De Palma, Claudia Moscheni, Elisabetta Catalani, Silvia Zecchini, Vincenzo Conte, Matteo Giovarelli, Sonia Caccia, Patrizia Procacci, Davide Cervia, Emilio Clementi, Cristiana Perrotta

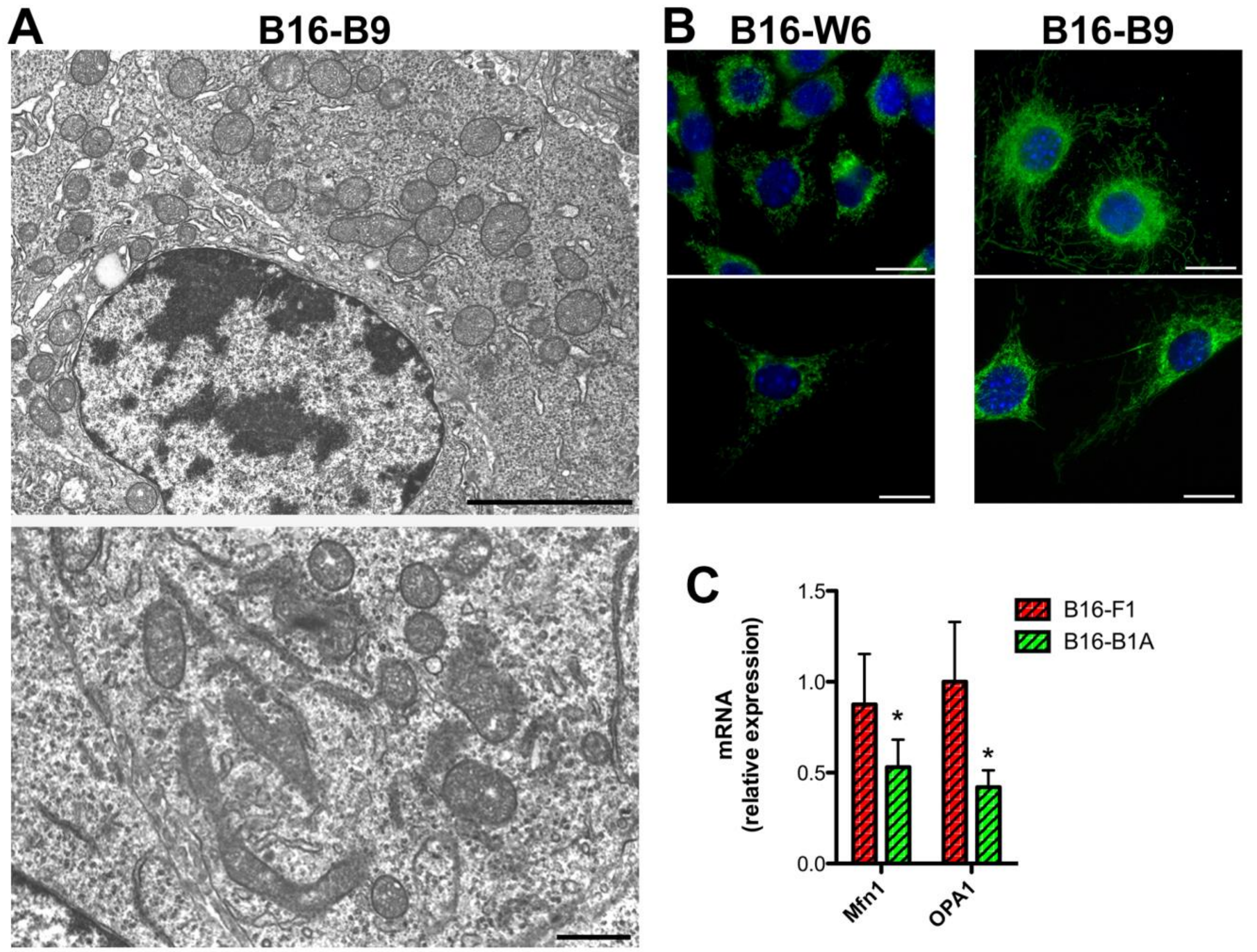

Supplementary Figure S1. A-SMase expression determines mitochondrial shape in vivo and in vitro. **A**. Transmission electron microscopy showing mitochondria in B16-B9 tumours. Upper panels scale bar = 5 µm. Lower panels scale bar = 1 µm. **B**. Representative fluorescence micrographs of cyclophylin f and Dapi staining of B16-B9 and B16-W6 cells. Scale bar = 20 µm. C. qPCR of Mfn1, and OPA1 on mRNA extract from B16-F1 and B16-B1A cells (n = 6). *p < 0.05 vs B16-F1.

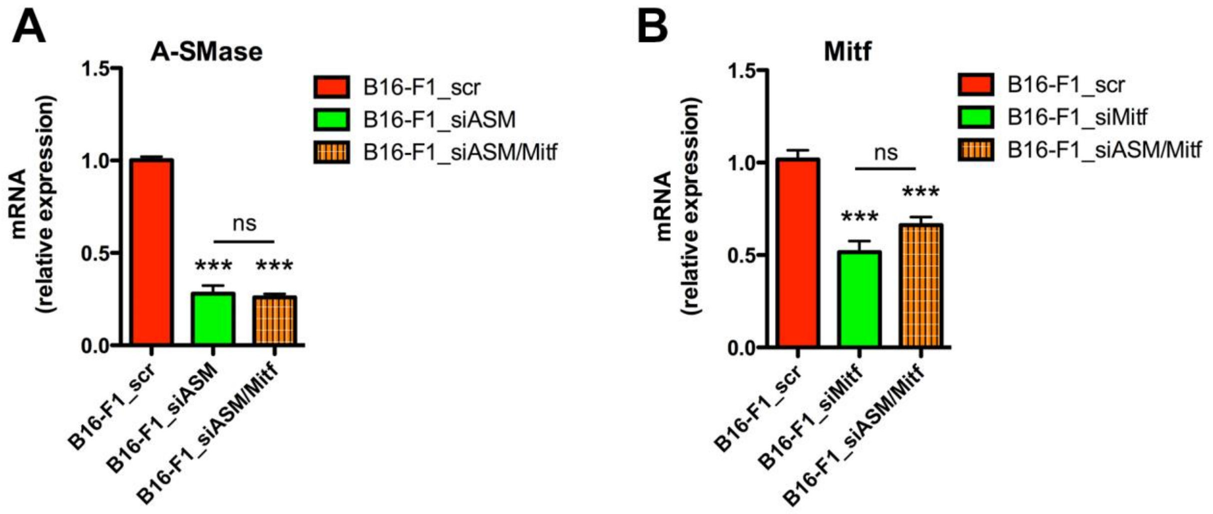

Supplementary Figure S2. Control of A-SMase and Mitf double knockdown. B16-F1 cells were transiently transfected with: the scrambled siRNA (B16-F1_scr), an A-SMase siRNA (B16-F1_siASM), a Mitf siRNA (B16-F1_siMitf) and a combination of the two siRNA (B16-F1_siASM/Mitf). **A**. A-SMase expression was evaluated by qPCR ($n \geq 6$). ***$p < 0.001$ vs B16-F1_scr. **B**. Mitf expression was evaluated by qPCR ($n \geq 6$). ***$p < 0.001$ vs B16-F1_scr.

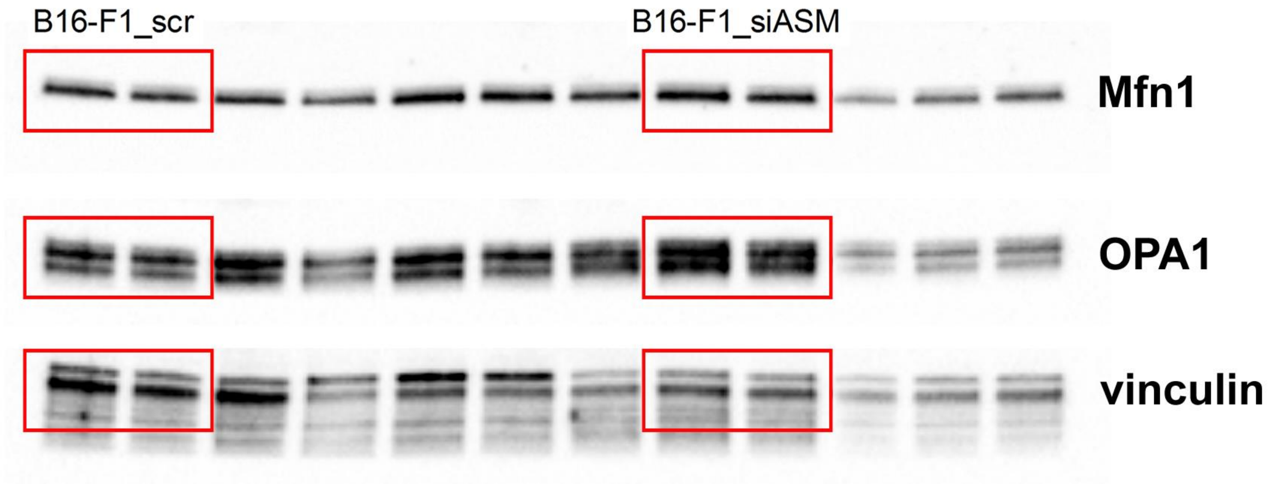

Supplementary Figure S3. Uncropped western blotting of the images shown in Figure 3B.
